# Antimicrobial Bamboo Materials Functionalized with ZnO and Graphene Oxide Nanocomposites

**DOI:** 10.3390/ma10030239

**Published:** 2017-02-27

**Authors:** Junyi Zhang, Bo Zhang, Xiufang Chen, Bingbing Mi, Penglian Wei, Benhua Fei, Xindong Mu

**Affiliations:** 1 Key Laboratory of Bio-Based Materials, Qingdao Institute of Bioenergy and Bioprocess Technology, Chinese Academy of Sciences, Qingdao 266101, China; zhangjy@qibebt.ac.cn (J.Z.); boews@sina.com (B.Z.); mome16@ sina.com (B.M.); muxd@qibebt.ac.cn (X.M.); 2 International Centre for Bamboo and Rattan, Beijing 100102, China; weipl01@sina.com

**Keywords:** ZnO, graphene oxide, bamboo, nanocomposites, antibacterial performance

## Abstract

Bamboo materials with improved antibacterial performance based on ZnO and graphene oxide (GO) were fabricated by vacuum impregnation and hydrothermal strategies. The Zn^2+^ ions and GO nanosheets were firstly infiltrated into the bamboo structure, followed by dehydration and crystallization upon hydrothermal treatment, leading to the formation of ZnO/GO nanocomposites anchored in the bulk bamboo. The bamboo composites were characterized by several techniques including scanning electron microscopy (SEM), Fourier transform infrared spectra (FTIR), and X-ray diffraction (XRD), which confirmed the existence of GO and ZnO in the composites. Antibacterial performances of bamboo samples were evaluated by the bacteriostatic circle method. The introduction of ZnO/GO nanocomposites into bamboo yielded ZnO/GO/bamboo materials which exhibited significant antibacterial activity against *Escherichia coli* (*E. coli*, Gram-negative) and *Bacillus subtilis* (*B. subtilis*, Gram-positive) bacteria and high thermal stability. The antimicrobial bamboo would be expected to be a promising material for the application in the furniture, decoration, and construction industry.

## 1. Introduction

With the growing demands for wood, paper, and other forest products globally, making the full exploitation and utilization of short-growth cycle herbaceous plants one of the best ways to solve the serious shortage of forest resources. Bamboo is the most promising woody herbaceous plant instead of wood because of its advantages, such as rapid growing, easy cultivation, high economic value, and ecological benefits. There are approximately 220,000 km^2^ of bamboo resources in China and the annual output of bamboo is probably to be 15–20 million tons [1]. Bamboo can be extensively used in the furniture, decoration, and construction industries for its beautiful figure, good mechanical properties, and excellent integrative performance like high strength, high density, and toughness. The maximum utility of bamboo as raw materials will reduce the over-dependency on wood, mitigate global warming and stimulate the forest industries. However, there are plentiful nutrients, including starch and protein, in the bamboo, making it easy to be eroded by moisture, sunlight, and fungi. Therefore, to prolong the service life of bamboo and economize natural resources, it is necessary to develop an effective method to achieve bamboo materials with excellent antibacterial activity. Regrettably, compared with wood, the work on bamboo modification and functionalization is still in its infancy.

In the last decades, much effort had been devoted to modify biomaterials to improve antibacterial activity. It was known that bacterial attachment on biomaterial surface is a key procedure during the development of infections. Modification of biomaterials by surface functionality using chemical reagents or nanomaterials was commonly applied. Environmental regulation has restricted the use of some toxic chemical reagents, like benzalkonium chloride, initiating a search for alternative modifiers. Compared with chemical reagents, inorganic materials, such as ZnO [2] and TiO_2_ [3], have advantages of low toxicity, low cost, chemical stability, and long effectiveness. Particularly, with an excited energy of 60 mV and a band gap of ~3.4 eV, ZnO has been used to effectively improve the wood performances such as UV-resistance [4], antibacterial activity [5,6] and thermal stability [7,8]. Tam et al. [5] reported that ZnO nanorods prepared by a facile method had shown good inhibition of bacteria reproduction on kinds of substrates. The low toxicity and antibacterial performance make ZnO a good choice for the modification of woody plants [9]. However, for the environmental protection, combining with other nanomaterials with better biocompatibility and non-toxicity will be helpful to further weaken the toxicity of Zn and enhance its antibacterial performance.

Recently, graphene oxide (GO) has attracted increasing attention in the chemical biology cross area due to its excellent chemical and physical properties such as good antimicrobial activity, outstanding biocompatibility, and non-toxicity. GO nanosheets have a large surface area with a two-dimensional structure and contains a variety of oxygen-containing groups. These abundant oxygen-containing groups make GO qualified, with good hydrophilic and outstanding biocompatibility properties [10,11]. For the particular construction, it also shows excellent mechanical and thermal stability properties [12]. All of these eminent advantages make it a desirable material in biomass applications. For instance, Shao et al. [13] previously found that a composite material comprising bacterial cellulose networks and GO nanosheets displayed an excellent antibacterial activity and good biocompatibility. Taking the full advantages of GO and ZnO, it is likely to enhance the performances of bamboo by combining ZnO nanoparticles with GO nanosheets. However, up to now, scarce reports have been focused on the bamboo modification by utilizing carbon-based nanomaterials.

Generally, surface modification is the most common method to improve the performance of biomaterials. The hierarchical structure and chemical composition of bamboo affords high possibilities for modification. The introduction of active materials into the bamboo cell wall structure would increase the connection between bamboo and inorganic materials, which would have a stronger impact on bamboo properties. There were little reports on bamboo modification by in situ synthesis. Recently, Merk et al. [14] illustrated a method of vacuum impregnation to introduce all precursors dissolved in water inside the wood, followed by an in situ hydrolysis reaction inter the matrix structure under a mild basic condition, consequently, CaCO_3_ was incorporated into the wood cell wall structure without changing its intrinsic microporous structure. The insertion of CaCO_3_ in bulk wood improved the mechanical property and thermal stability more effectively than surface modification. Thus, through this strategy, inorganic materials might be fixed more stable in the bulk bamboo which were probably beneficial to generate better antibacterial property. Herein, we modified bamboo by ZnO nanoparticles and GO nanosheets through the method of vacuum assisted impregnation under ultrasound treatment and hydrothermal process. The ZnO nanoparticles and GO nanosheets were probably incorporated deeply into the bamboo structure to form a hybrid material at the macromolecular level. The antibacterial properties of functionalized bamboo were evaluated by using *Escherichia coli* (*E. coli*, Gram-negative) and *Bacillus subtilis* (*B. subtilis*, Gram-positive) bacteria. The functionalization with ZnO and GO nanocomposites were found to be effective in improving the antibacterial properties of bamboo. Meanwhile, a thermogravimetric (TG) analyzer was also used to examine the effects of the modifiers on the thermal properties. This work would provide an effective approach to improve the properties of bamboo and make it a promising material for the application in the furniture, decoration, and construction industries.

## 2. Materials and Methods

Raw materials of bamboo were obtained from Anhui Taiping test center of the International Centre for Bamboo and Rattan. The exterior and interior layers of the bamboo were removed, and then they were cut into blocks of 10 mm (longitudinal) × 10 mm (tangential) × 10 mm (radial) in size. All blocks were ultrasonically washed with deionized water and alcohol for 30 min at room temperature, respectively. To further remove impurity inside the bamboo structure, all of the samples were impregnated in vacuum under the water for several times and then dried for 24 h at 60 °C under vacuum for further use. The as-prepared bamboo was named as original bamboo (OB). Additionally, a GO nanosheet suspension (10 g·L^−1^) prepared by Hummer’s method was purchased from Qingdao Huagao Moxi science and technology Co., LTD (Qingdao, China). The other chemicals of analytical pure were all supplied by Sinopharm Chemical Reagent Co., Ltd. (Shanghai, China). All of them were used as received without further purification.

### 2.1. Preparation of Bamboo Composites

Firstly, bamboo blocks were pre-treated by an ultrasound and vacuum-assisted impregnation method. During this stage, a GO suspension was added into the saturated solutions of zinc acetate to form a homogeneous mixture with a GO concentration of 1.67 g·L^−1^, and the bamboo blocks were immersed into the above mixture. Then the mixture was ultrasonic-treated and vacuum- impregnated for several times to diffuse GO nanosheets and Zn^2+^ ions into the porous bamboo structure to a great extent. After that, the pH value of the mixture was adjusted to 10 by adding a monoethanolamine solution (MEA, 1 mol·L^−1^). Then the blocks were soaked for 12 h. Finally, the samples which were removed from the suspension and dried at 60 °C in vacuum were used for further preparation.

Secondly, 1.5 mL of GO suspension (10 g·L^−1^) was added into 20 mL zinc nitrate solution (0.05 mol·L^−1^) and treated by ultrasonic for 0.5 h. The pH value was adjusted to 10 by adding MEA solution (1 mol·L^−1^) and the mixture was stirred to form homogenous suspension. Afterwards, the as-prepared bamboo samples were cast into the above solution, and then transferred into a Teflon-lined stainless-steel autoclave. The hydrothermal reaction was performed at a temperature of 120 °C for 3 h. Then the resulting products were washed repeatedly by deionized water to remove redundant Zn^2+^ ions and GO nanosheets. Finally, the as-made samples of ZnO/GO/bamboo were dried at 60 °C for 24 h under vacuum to remove the water until constant weight. For comparison, GO/bamboo and ZnO/bamboo were synthesized by the same method. The ZnO/GO/bamboo, GO/bamboo, and ZnO/bamboo were named as ZGB, GO, and ZB, respectively.

### 2.2. Characterization

To obtain the surface morphologies and the content of zinc, the composites were investigated by using transmission electron microscopy (TEM, Hitachi, H-7650, Tokyo, Japan) and scanning electron microscopy (SEM, Hitachi, S-4800) combined with an energy dispersive X- analysis system. The chemical compositions of the bamboo were tested by the Fourier transform infrared spectroscopy (FTIR, Nicolet 6700, Waltham, MA, USA). A X-ray diffraction (XRD) instrument (Bruker, D8 Advance, Karlsruhe, Germany) was used to carry out the XRD measurements by utilizing Cu Kα1 radiation in the 2θ range of 5°–60°. The thermal performances of the samples were investigated by a TG analyzer (Q600, TA Instruments, New Castle, DE, USA) from 25 °C to 1000 °C under a nitrogen atmosphere with a heating rate of 10 °C·min^−1^. The release of zinc ions was tested by inductively-coupled plasma mass spectrometry (ICP-Ms, Thermo, ICAP-QC, Waltham, MA, USA).

### 2.3. Antibacterial Test

In this work, the bacteriostatic circle method was adopted for antibacterial testing [15]. The outcomes were estimated by their growth inhibition properties against Gram-negative bacteria (*E. coli*) and Gram-positive bacteria (*B. subtilis*). Four microliters of *E. coli* and 4 µL *B. subtilis* were removed and then cultivated in 10 mL culture medium, respectively. After cultivation for 24 h in a sterile incubator, 100 µL *E. coli* or 100 µL *B. subtilis* was transferred to the solid medium in a sterile environment, and the solution was spread by a disinfected glass rod to cover the surface evenly. Then the bamboo block samples (OB, ZB, GB, ZGB) were fixed on the culture dishes coated with *E. coli* or *B. subtilis*. Finally, the dishes were put into the incubator to culture for 24 h at 37 °C. The antibacterial performance was estimated by the size of inhibition zone.

## 3. Results and Discussion

### 3.1. Synthesis and Characterization of Bamboo Composites

**Figure 1:**
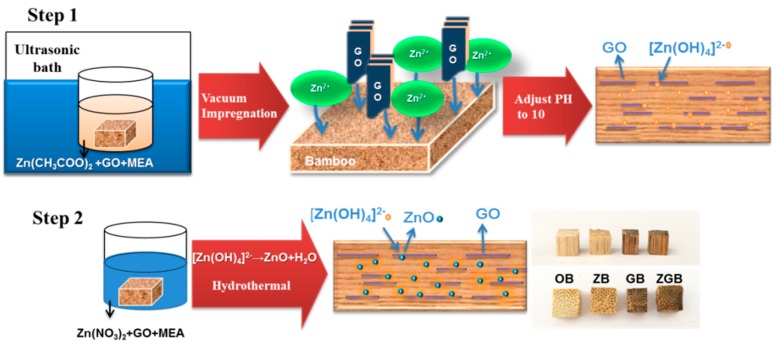
Schematic illustration for preparing ZGB by using the impregnation and hydrothermal processes.

Figure 1 presents the schematic illustration of the fabrication of bamboo composite samples. The parenchyma and vascular tissues of bamboo constitute a porous and tubular structure. The structure provides continuous transport pathways for the Zn^2+^ ions and GO nanosheets effectively. In this work, Moso bamboo blocks wiped of their exterior and interior layer were used as the origin materials. After pre-treatment with ultrasonically washing, they were all immersed into a mixed suspension of GO nanosheets and Zn(CH_3_COO)_2_. Under the ultrasonic treatment, Zn^2+^ ions were easily bound to oxygen-containing groups on GO nanosheets by electrostatic interactions and coordination reaction. By vacuum-assisted impregnation, Zn^2+^/GO nanosheets and Zn^2+^ were probably diffused in depth into the porous bamboo structure and attached in and/or the surface of the cell wall. With the dropwise addition of MEA solution to adjust pH value to 10, Zn^2+^ reacted with OH^−^ and formed the [Zn(OH)_4_]^2−^ units inside the bamboo. Under hydrothermal treatment, [Zn(OH)_4_]^2−^ dehydrated into ZnO nuclear and grew bigger gradually [16]. Meanwhile, hydrothermal reaction provided a high temperature and pressure environment which also facilitated more GO nanosheets spread into the porous structure of bamboo and formed interconnected structure through hydrogen bonds between the carbonyl groups of GO and the hydroxyl groups of bamboo. The GO nanosheets inside the bamboo were also anchored by ZnO nanoparticles homogeneously under hydrothermal condition to form ZnO/GO nanocomposites.

The weight percent gains of bamboo composites were studied in detail. For the control original bamboo through hydrothermal treatment with distilled water under similar condition, the bamboo exhibited a weight loss of 7.9 wt % which was mainly due to the leaching of the extractives and impurities. After modified with ZnO nanoparticles or GO nanosheets alone, the bamboo displayed a weight gain of 2.2 and 1.0 wt %, respectively, compared to the control bamboo. The weight gain was directly related to the ZnO or GO content, suggesting that ZnO or GO was incorporated into the bamboo. For the ZGB sample, the composites showed a weight gain of 3.1 wt %, revealing the total contents of ZnO and GO in the composites. Some control experiments were also carried out to confirm the existence of ZnO in the ZB and ZGB samples. OB, ZB, and ZGB samples were calcined at 800 °C in air for 2 h. As organic components were burn thoroughly in such high temperature and only inorganic residues were left. The amount of inorganic residue for OB, ZB and ZGB samples were 0.6, 3.1 and 1.8 wt %, respectively. Thus, the ZnO content in the ZB and ZGB was calculated to be 2.5 and 1.2 wt %, respectively.

**Figure 2:**
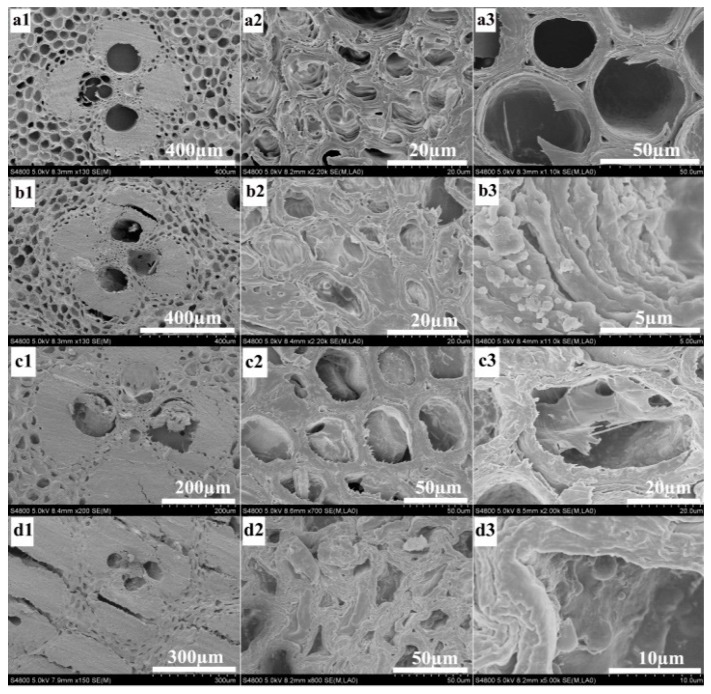
SEM images of OB, GB, ZB, and ZGB composites: OB (**a1**–**a3**); ZB composite (**b1**–**b3**); GB composite (**c1**–**c3**); ZGB composite (**d1**–**d3**).

**Figure 3:**
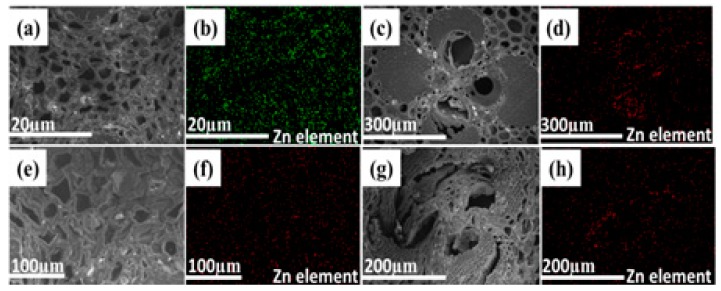
SEM and mapping images of ZB (**a**–**d**) and ZGB composites (**e**–**h**).

The SEM was utilized to detect the changes of bamboo morphologies before and after the modification (Figure 2). The distribution of Zn element in modified bamboo blocks at cross-sections was also examined by mapping analysis (Figure 3). The transverse of bamboo samples were cut off a depth of about 2 mm and then cut into ultrathin pieces for SEM analysis. Figure 2 showed the morphology of bamboo samples. Figure 2(a1–a3) exhibited the microstructure of OB sample. It is obviously observed that different forms of vascular bundle and parenchymatous tissues with empty spaces dispersed regularly inside the bamboo. Figure 2(b1–b3) displayed that ZnO granular were distributed along the surface of vessel in the ZB. Figure 3a–d showed SEM images and corresponding Zn elemental mapping of ZB at cross sections. The mapping images of ZB also exhibited a uniform distribution of Zn element in the bamboo with a weight content of 3.0 wt %. Figure 2(c1–c3) showed GO was penetrated into the vessel through vacuum impregnation and hydrothermal method. For ZGB composites shown in Figure 2(d1–d3) and Figure 3e–h, Zn elements were also distributed well inside the bamboo with a weight content of 0.9 wt %. It was worth mentioning that all samples displayed a similar arrangement of network structure, suggesting that the bamboo structure was retained well during treatment.

**Figure 4:**
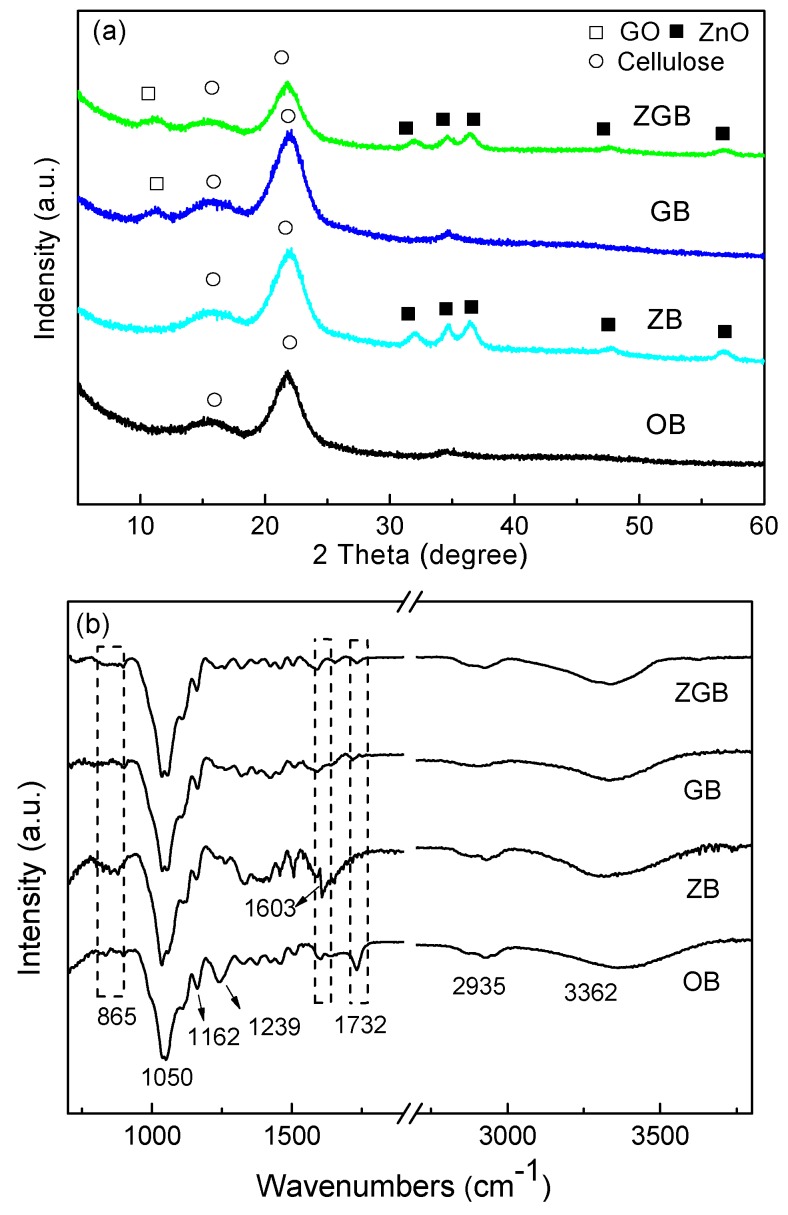
XRD pattern (**a**) and FTIR spectra (**b**) of bamboo samples (OB, ZB, GB, and ZGB).

XRD was used to estimate the elemental compositions of the bamboo samples. The results were showed in Figure 4a. For all the bamboo samples, similar peaks at about 15° and 22° attributed to (101) and (002) planes of the typical structure of cellulose were all observed [17,18]. Apart from the characteristic peaks of cellulose, five new peaks at around 32°, 34°, 36°, 47°, and 56° were found in the ZB sample, assigned to (100), (002), (101), (110), and (102) planes of the wurtzite-type ZnO (JCPDS: 36-1451), respectively [19]. The size of ZnO particles in the ZB was about 8.0 nm, calculated from Scherrer equation. For the GB sample, the additional diffraction peak approximately at 10° was attributed to the (001) peak of GO [20,21]. The XRD pattern of ZGB also showed typical five peaks of wurtzite-type ZnO and (001) peak of GO, confirming the existence of ZnO and GO in the bamboo composites. The particle size of ZnO inside ZGB sample was approximately 6.1 nm.

Figure 4b showed the FTIR spectra of ZGB within the range of 500–4500 cm^−1^, with OB, ZB, and GB for comparison. For OB, the broad peak around 3150–3600 cm^−1^ was assigned to the intramolecular hydrogen bond and the hydroxyl groups [22,23]. The peak at 1162 cm^−1^ was in accordance with the C–O asymmetric bridge stretching and the peak at 1050 cm^−1^ corresponded to the alkoxy group vibration [24]. Additionally, the small sharp peak at 1732 cm^−1^ was due to the stretching of C=O which commonly appeared in carbonyls and ester groups of hemicellulose. Other peaks at 1239 cm^−1^ and 2935 cm^−1^ were assigned to C–OH stretching and C–H asymmetry of epoxy groups, respectively [25,26]. For ZB, GB, and ZGB composites, most of the characteristic peaks in the spectra were similar to OB except for some small differences. In the case of ZB, the stretching vibrations n the ZB spectra at 1603 cm^−1^ represented the intramolecular hydrogen bonds, and the peak at around 865 cm^−1^ was related to metal oxide bonding, which further indicated the existence of ZnO [27]. In ZGB plot, the peak positioned at 1730 cm^−1^ showed the C=O stretching vibration of GO or hemicellulose, as both of them has the characteristic peaks of carbonyls and ester groups [28]. Combined with XRD results, the peak at 1730 cm^−1^ might be the combined result of bamboo hemicellulose and GO. So it can be speculated that GO sheets might be effectively incorporated in the bamboo.

### 3.2. Thermal Stability

**Figure 5:**
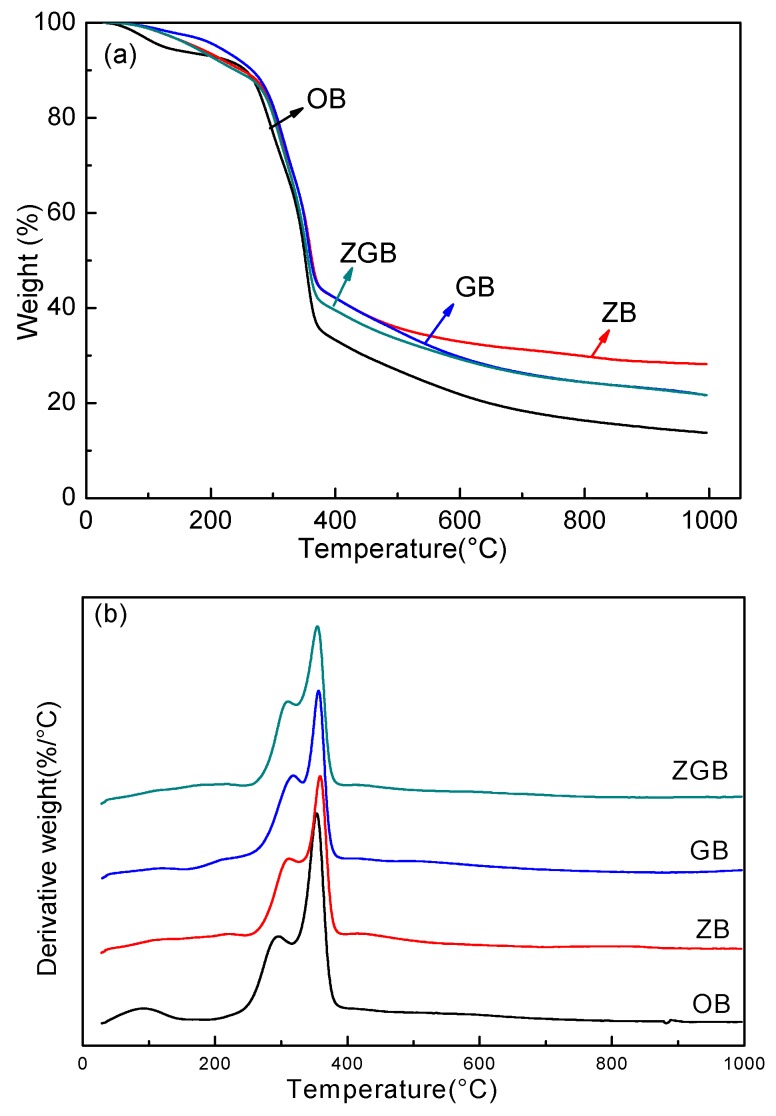
TG (**a**) and DTG (**b**) curves of bamboo samples (OB, ZB, GB, ZGB), respectively.

Figure 5 presented the TG and DTG curves of bamboo samples (OB, ZB, GB, ZGB). Compared with OB, the TG curves of ZB, GB, and ZGB were shifted to higher temperature, suggesting better thermal stability. The first weight losses of all the samples were slight, in the range from 20 °C to 190 °C for the loss of physically-adsorbed and loosely hydrogen-bound water molecules before decomposition [29]. Another three stages of the pyrolysis were clearly displayed in the TG curves. For the ZGB sample, the degradation of hemicellulose happened at 190–320 °C with a low weight loss of approximately 20.8%. The degradation of cellulose happened at 320–400 °C with a weight loss of 57.9%. In the last stage (400–1000 °C), all of the components in the bamboo samples degraded, except the lignin due to its high decomposition temperature, and there were no other obvious characteristic exothermic stages. Similarly, there were two sharp peaks in DTG curves shown in Figure 5b. According to TG curves of ZGB, the first thermal decomposition occurred at about 296 °C, which was assigned to the pyrolysis of hemicellulose, and the contiguous peak approximately at 353 °C was attributed to the decomposition of cellulose [30,31]. These results implied that the thermal stability of the treated bamboo samples were higher than OB due to the modification of ZnO and GO.

### 3.3. Antibacterial Properties

**Figure 6:**
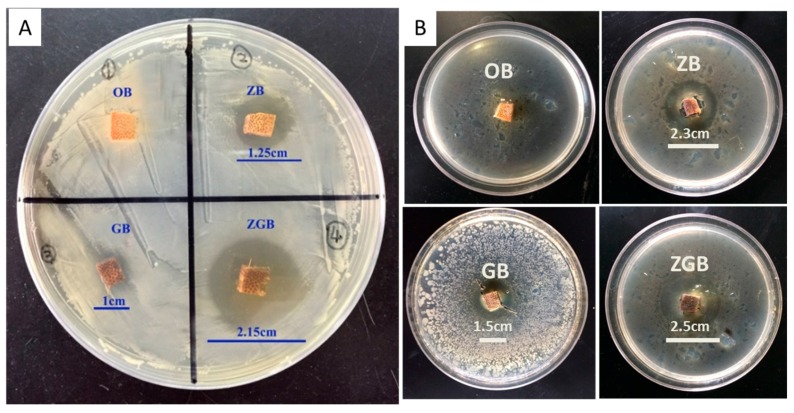
Antibacterial activities of bamboo samples (OB, ZB, GB, and ZGB) against *E. coli* (**A**) and *B. subtilis* (**B**).

The antibacterial properties of the control sample (OB) and the composites (ZB, GB, ZGB) were qualitatively investigated by the bacteriostatic circle method against one Gram-negative (*E. coli*) and one Gram-positive (*B. subtilis*), and the results were shown in Figure 6. Figure 6A displayed the antibacterial activities of bamboo samples against *E. coli*. It can be observed that OB had little antibacterial activity without an inhibition zone. By contrast, ZB and GB could inhibit the bacterial growth to some extent with the inhibition zone of 1.25 and 1 cm, respectively. The results indicated the effective antibacterial activity of ZnO particles and GO nanosheets for Gram-negative bacteria. Notably, GB showed obvious inhibition zone against *E. coli* in the antibacterial test, which illustrated that some GO nanosheets on the surface of GB sample were diffused into the culture medium. This might be due to the relative high content of GO nanosheets with small size on the surface of GB sample, and their diffusion into the surrounding culture medium. The presence of GO nanosheets could insulate bacteria from growth environment [32,33] and, thus, resulted in inhibition zone around GB sample. Compared with Zn^2+^, GO was more difficult to spread in the culture medium, so ZB showed larger inhibition zone against *E. coli* than that of GB. As ZnO and GO were co-incorporated into the bamboo, the hybrid materials displayed significantly improved inhibition effect against *E. coli* resulting in the inhibition zone of 2.15 cm. Figure 6B showed the antibacterial activities of bamboo samples against *B. subtilis*. Compared with OB which had little antibacterial ability with no inhibition zone, ZB, GB, and ZGB samples showed obvious inhibition zone of 2.3, 1.5 and 2.5 cm, respectively. Combined with the above results, the ZGB was demonstrated to be excellent antibacterial materials for both Gram-positive and Gram-negative bacteria.

**Figure 7:**
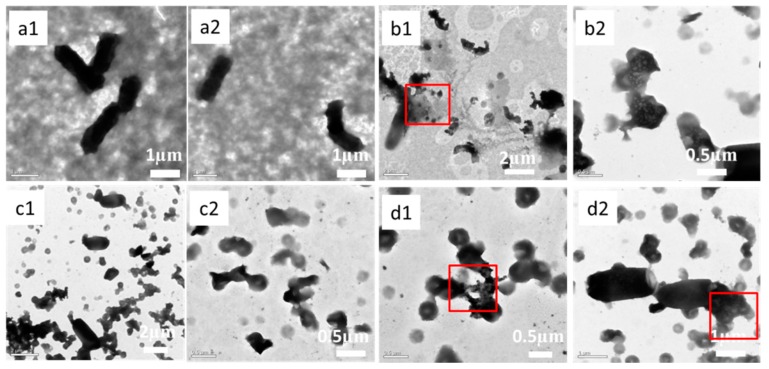
TEM images of *E. coli* mixed with bamboo samples after 12 h: OB (**a1**,**a2**), ZB (**b1**,**b2**), GB (**c1**,**c2**), and ZGB (**d1**,**d2**).

TEM was used to investigate the interaction between bacteria and bamboo samples (OB, ZB, GB, and ZGB). *E. coli*, which could be test conveniently without staining, was chosen for the TEM examination. Firstly, *E. coli* solution was diluted with sterilized water until the optical density of the diluted bacteria solution was between 0.7 and 0.8 at 600 nm. Next, the bamboo samples (OB, ZB, GB, or ZGB) were cut off a ~1 mm thickness from the surface and crushed into chippings, and 1 mL *E. coli* solution was mixed with 6 mg chippings. Then the mixture was shaking at 25 °C for 10 min. Finally, a drop of the supernatant was dropped on the copper-grid and dried in air for 12 h before TEM test. The images of TEM were shown in Figure 7. In comparison with the results from OB, ZB, GB, and ZGB system, it can be seen clearly that the morphology of *E. coli* treated with ZB, GB and ZGB samples changed significantly, compared to OB treated bacteria. The OB treated *E. coli* showed a rod-like shape (see Figure 7(a1,a2)), similar to the pristine bacteria. When ZB was used to treat *E. coli*, the bacteria cell structure was destroyed evidently, and cytoplasm was shed out from the cell after exposed to ZB for 12 h (see Figure 7(b1,b2)), which indicated that ZnO could interact with the membrane of *E. coli* and inhibit their growth eventually [34,35]. For GB system (see Figure 7(c1,c2)), the *E. coli* were disrupted into small fragments, which might be due to the insulation of bacteria from growth environment and mechanical cutting [28,36]. In the case of ZGB (see Figure 7(d1,d2)), the *E. coli* were changed from rod-shaped to an indefinite shape, accompanied with the leakage of cytoplasm (shown in red square in Figure 7d) and the disintegration of the bacteria. Combined with the results of ZB and GB, ZGB composites might lead to a cessation of growth and bacterial death because of the synergistic effect of ZnO and GO.

ZnO nanoparticles had been reported to be effective antibacterial agent. Combined with the results of TEM and the antibacterial test, the antibacterial performance of ZnO might be owing to the formation of peroxides or the release of Zn^2+^ ions, which would interact with the bacterial cell walls and membranes harmfully and inhibit their growth eventually [34,35]. Moreover, the presence of GO nanosheets could insulate bacteria from the growth environment [32,33] and damage bacteria cell membranes by mechanical cutting [28,36]. The formation of a hydrogen bond between the hydroxyl groups of bamboo and carbonyl groups of GO nanosheets would further strengthen the stability of antibacterial properties [36]. In addition, GO could effectively decrease the surface free energy of the sample [13], which also led to the reduction of bacterial attachment to the samples. Combining the advantages of ZnO particles and GO nanosheets, GO nanosheets in the bamboo also offered supports for anchoring ZnO nanoparticles, which might allow effective attachment of ZnO nanoparticles on the surface of bacteria, leading to the increase of the opportunity for contact between ZnO nanoparticles and bacteria [36,37]. As a result, the modification of ZnO and GO nanosheets could enhance antibacterial activity of bamboo significantly.

To support the mode of action, the release of Zn^2+^ ions from the ZGB composites in liquid culture medium were also measured by ICP-Ms [38]. Firstly, the exterior layer (about 2 mm thickness) of ZGB was scraped off, crushed into chipping,s and mixed with 1 mL liquid culture medium (bacteria-free), followed by shaking in a shaker at 37 °C for 24 h. After that, 600 μL of the mixtures was taken out and centrifuged (8000 rpm × 15 min) to remove the insoluble residues. Finally, the supernatant was collected and dealt with HNO_3_/H_2_O_2_ (1:1, *v*/*v*) at 100 °C for 15 min and diluted with 2 mL 2% HNO_3_ for ICP-Ms measurements. The ICP results showed the concentration of Zn^2+^ ions in the final diluted mixture was 8.1 mg·L^−1^, which confirmed that small amount of Zn^2+^ ions indeed were diffused in the medium. Combined with the results of TEM (see Figure 7), it could be further proved that Zn^2+^ ions in the ZGB sample would be released and interacted with the bacterial cell walls and membrane of *E. coli*, finally resulting in bacterial death.

## 4. Conclusions

In summary, we employed ZnO nanoparticles and GO nanosheets by a facile method for the modification of bamboo. ZGB was prepared through vacuum impregnation and hydrothermal processes, and ZnO nanoparticles and GO nanosheets were successfully incorporated in the bamboo, probably forming a hybrid material at the macromolecular level. Compared with the modification of ZnO nanoparticles or GO nanosheets alone, the combination of ZnO nanoparticles and GO nanosheets endowed the bamboo with better performances in its antibacterial properties. Moreover, the ZGB composites also displayed high thermal stability. This work provided an interesting and effective approach to prolong the service life of bamboo.

## References

[1] Liu Z.J., Jiang Z.H., Fei B.H., Cai Z.Y., Liu X.E., Yu Y. (2012). Bamboo pellets: A potential and commercial pellets in China. Sci. Silvae Sin..

[2] Feng X., Feng L., Jin M., Zhai J., Jiang L., Zhu D. (2004). Reversible super-hydrophobicity to super-hydrophilicity transition of aligned ZnO nanorod films. J. Am. Chem. Soc..

[3] Chen X.F., Wang X.C., Fu X.Z. (2009). Hierarchical macro/mesoporous TiO_2_/SiO_2_ and TiO_2_/ZrO_2_ nanocomposites for environmental photocatalysis. Energy Environ. Sci..

[4] Li J.P., Sun Q.F., Yao Q.F., Wang J., Han S.J., Jin C. (2015). Reversibly light-switchable wettability between super hydrophobicity and superhydrophilicity of hybrid ZnO/bamboo surfaces via alternation of UV irradiation and dark storage. Prog. Org. Coat..

[5] Tam K.H., Djurišić A.B., Chan C.M.N., Xi Y.Y., Tse C.W., Leung Y.H., Chan W.K., Leung F.C.C., Au D.W.T. (2008). Antibacterial activity of ZnO nanorods prepared by a hydrothermal method. Thin Solid Films.

[6] Jiang Y.H., Zhang L.L., Wen D.S., Ding Y.L. (2016). Role of physical and chemical interactions in the antibacterial behavior of ZnO nanoparticles against *E. coli*. Mater Sci. Eng. C.

[7] Scharber M.C., Mühlbacher D., Koppe M., Denk P., Waldauf C., Heeger A., Brabec C. (2006). Design rules for donors in bulk-heterojunction solar cells-towards 10% energy-conversion efficiency. Adv. Mater..

[8] Wei X.Q., Zhang Z., Yu Y.X., Man B.Y. (2009). Comparative study on structural and optical properties of ZnO thin films prepared by PLD using ZnO powder target and ceramic target. Opt. Laser Technol..

[9] Choi O., Deng K.K., Kimc N.J., Loss R., Surampalli R.Y., Hu Z.Q. (2008). The inhibitory effects of silver nanoparticles, silver ions, and silver chloride colloids on microbial growth. Water Res..

[10] Li Y.Q., Umer R., Samad Y.A., Zheng L.X., Liao K. (2013). The effect of the ultrasonication pre-treatment of graphene oxide (GO) on the mechanical properties of GO/polyvinyl alcohol composites. Carbon.

[11] Katsnelson M.I., Novoselov K.S. (2007). Graphene: New bridge between condensed matter physics and quantum electrodynamics. Solid State Commun..

[12] Balandin A.A., Ghosh S., Bao W.Z., Calizo I., Teweldebrhan D., Miao F., Lau C.N. (2008). Superior thermal conductivity of single-layer grapheme. Nano Lett..

[13] Shao W., Liu H., Liu X.F., Wang S.X., Zhang R. (2015). Anti-bacterial performances and biocompatibility of bacterial cellulose/graphene oxide composites. RSC Adv..

[14] Gkikas M., Theodosopoulos G.V., Das B.P., Tsianou M., Iatrou H., Sakellariou G. (2014). Gold-decorated graphene nanosheets composed of a biocompatible non-charged water-soluble polypeptide. Eur. Polym. J..

[15] Vijayaprasath G., Murugan R., Palanisamy S., Prabhu N.M., Mahalingam T., Hayakaw Y., Ravi G. (2016). Role of nickel doping on structural, optical, magnetic properties and antibacterial activity of ZnO nanoparticles. Mater. Res. Bull..

[16] Raula M., Rashid M.H., Paira T.K., Dinda E., Mandal T.K. (2010). Ascorbate-assisted growth of hierarchical ZnO nanostructures: Sphere, Spindle, and flower and their catalytic properties. Langmuir.

[17] Chen X., Zhou S.K., Zhang L.M., You T.T., Xu F. (2016). Adsorption of heavy metals by graphene oxide/cellulose hydrogel prepared from NaOH/urea aqueous solution. Materials.

[18] Yan Z., Chen S., Wang H., Wang B., Jiang J. (2008). Biosynthesis of bacterial cellulose/multi-walled carbon nanotubes in agitated culture. Carbohydr. Polym..

[19] Yang S., Chen F., Shen Q., Lavernia E.J., Zhang L.M. (2016). Microstructure and electrical properties of AZO/graphene nanosheets fabricated by spark plasma sintering. Materials.

[20] Fu L., Lai G.S., Zhang H.L., Yu A.M. (2015). One-pot synthesis of multipod ZnO-carbon nanotube-reduced graphene oxide composites with high performance in photocatalysis. J. Nanosci. Nanotechnol..

[21] Xu S.H., Li F., Pham T.S.H., Yu A.M., Han F.G., Chen L. (2015). Preparation of ZnO flower/reduced graphene oxide composite with enhanced photocatalytic performance under sunlight. Ceram. Int..

[22] Ullah M.W., Ul-Islam M., Khan S., Kim Y., Park J.K. (2016). Structural and physico-mechanical characterization of bio-cellulose produced by a cell-free system. Carbohydr. Polym..

[23] Feng Y., Zhang X., Shen Y., Yoshino K., Feng W. (2012). A mechanically strong, flexible and conductive film based on bacterial cellulose/graphene nanocomposite. Carbohydr. Polym..

[24] Sun D., Yang J., Wang X. (2010). Bacterial cellulose/TiO_2_ hybrid nanofibers prepared by the surface hydrolysis method with molecular precision. Nanoscale.

[25] Wang S.L., Wang C.Y., Liu C.Y., Zhang M., Ma H., Li J. (2012). Fabrication of superhydrophobic spherical-like α-FeOOH films on the wood surface by a hydrothermal method. Coll. Surf. A.

[26] Satheesh K., Jayavel R. (2013). Synthesis and electrochemical properties of reduced graphene oxide via chemical reduction using thiourea as a reducing agent. Mater. Lett..

[27] Yazhini K.B., Prabu H.G. (2015). Study on flame-retardant and UV-protection properties of cotton fabric functionalized with ppy–ZnO–CNT nanocomposite. RSC Adv..

[28] Hu W.B., Peng C., Luo W.J., Lv M., Li X.M., Li D., Huang Q., Fan C.H. (2010). Graphene-based antibacterial paper. ACS Nano.

[29] Yang J.Z., Sun D.P., Li J., Yang X.J., Yu J.W., Hao Q.L., Liu W.M., Liu J.G., Zou Z.G., Gu J. (2009). In situ deposition of platinum nanoparticles on bacterial cellulose membranes and evaluation of PEM fuel cell performance. Electrochim. Acta.

[30] Yang H., Yan R., Chen H., Lee D.H., Zheng C. (2007). Characteristics of hemicellulose, cellulose and lignin pyrolysis. Fuel.

[31] Li X.B., Zhang X., Li L.C., Huang L.L., Zhang W., Ye J.D., Hong J.G. (2016). Preparation of nano-ZnO/regenerated cellulose composite particles via co-gelation and low-temperature hydrothermal synthesis. Mater. Lett..

[32] Akhavan O., Ghaderi E., Esfandiar A. (2011). Wrapping bacteria by graphene nanosheets for isolation from environment, reactivation by sonication, and inactivation by near-infrared irradiation. J. Phys. Chem. B.

[33] Akhavan O., Ghaderi E. (2010). Toxicity of graphene and graphene oxide nanowalls against bacteria. ACS Nano.

[34] Sawai J., Microbiol J. (2003). Quantitative evaluation of antibacterial activities of metallic oxide powders (ZnO, MgO and CaO) by conductimetric assay. Methods.

[35] Brayner R., Ferrari-Iliou R., Brivois N., Djediat S., Benedetti M.-F., Fievet F. (2006). Toxicological impact studies based on *Escherichia coli* bacteria in ultrafine ZnO nanoparticles colloidal medium. Nano Lett..

[36] Achaby M.E., Essamlali Y., Miri N.E., Snik A., Abdelouahdi K., Fihri A., Zahouily M., Solhy A. (2014). Graphene oxide reinforced chitosan/ polyvinylpyrrolidone polymer bio-nanocomposites. J. Appl. Polym. Sci..

[37] Chen J.N., Peng H., Wang X.P., Shao F., Yuan Z.D., Han H.Y. (2014). Graphene oxide exhibits broad-spectrum antimicrobial activity against bacterial phytopathogens and fungal conidia by intertwining and membrane perturbation. Nanoscale.

[38] Wang Y.W., Cao A.N., Jiang Y., Zhang X., Liu J.H., Liu Y.F., Wang H.F. (2014). Superior antibacterial activity of zinc oxide/graphene oxide composites originating from high zinc concentration localized around bacteria. ACS Appl. Mater. Interfaces.

